# Psychiatric rating scales in Urdu: a systematic review

**DOI:** 10.1186/1471-244X-7-59

**Published:** 2007-10-26

**Authors:** Syed Ahmer, Rafey A Faruqui, Anita Aijaz

**Affiliations:** 1 Department of Psychiatry, The Aga Khan University, Stadium road, Karachi-74800, Pakistan; 2 National Centre for Brain Injury Rehabilitation, St. Andrew's Hospital, Northampton NN1 5DG UK

## Abstract

**Background:**

Researchers setting out to conduct research employing questionnaires in non-English speaking populations need instruments that have been validated in the indigenous languages. In this study we have tried to review the literature on the status of cross-cultural and/or criterion validity of all the questionnaires measuring psychiatric symptoms available in Urdu language.

**Methods:**

A search of Medline, Embase, PsycINFO and  was conducted using the search terms; Urdu psychiatric rating scale, and Urdu and Psychiatry. References of retrieved articles were searched. Only studies describing either cross-cultural or criterion validation of a questionnaire in Urdu measuring psychiatric symptoms were included.

**Results:**

Thirty two studies describing validation of 19 questionnaires were identified. Six of these questionnaires were developed indigenously in Urdu while thirteen had been translated from English. Of the six indigenous questionnaires five had had their criterion validity examined. Of the thirteen translated questionnaires only four had had both their cross-cultural and criterion validity assessed.

**Conclusion:**

There is a paucity of validated questionnaires assessing psychiatric symptoms in Urdu. The BSI, SRQ and AKUADS are the questionnaires that have been most thoroughly evaluated in Urdu.

## Background

With an estimated population of about 165 million [1] Pakistan is the sixth most populous nation in the world. Although only about 8% of Pakistanis speak Urdu (the national language of Pakistan) as their first language, most people in Pakistan are bilingual speaking their regional language and Urdu almost equally easily [2]. English is spoken mostly by the educated classes and used for official correspondence. With a literacy ratio of 44% and about 50% of the population receiving only primary or below primary education [3] there are many Pakistanis who are unable to read and understand English.

Most psychiatric research involves use of questionnaires of one sort of another. Most of these questionnaires have been developed in the English language and Western culture. There are questions as to how applicable or relevant these questionnaires would be in a primarily non-English speaking Eastern nation like Pakistan. We, therefore, need questionnaires that are in a language that can be understood by majority of the Pakistani people, like Urdu, and are relevant to their culture. In the absence of such questionnaires the two options available are either to create a new questionnaire in Urdu, or to translate and adapt an already established questionnaire from English.

If the latter route of translating and adapting an established questionnaire is taken, which is more often taken by virtue of being a less daunting task than creating a new questionnaire, there are five major domains of cross-cultural validity that need to be considered [4,5].

1. Content validity. The content of the instrument should be relevant in the culture into which the instrument is being translated.

2. Semantic validity. The words in the original instrument and the translated instrument should have the same meaning.

3. Technical validity. The method of assessment is comparable in each culture e.g. self-rated instruments assume literacy which is not very high in Pakistan.

4. Criterion validity. The interpretation of responses to similar items in source and target languages should remain the same when compared with the norm of each culture studied.

5. Conceptual validity. The instrument is measuring the same theoretical construct within each culture.

Whether indigenously developed or translated, all new questionnaires also need to have their criterion validity established against an existing gold standard in an appropriate group of respondents to be declared clinically useful [6]. In this manner their validity coefficients such as sensitivity, specificity, positive predictive value and negative predictive value can be established to make them comparable with other similar questionnaires. An instrument is valid if it correctly identifies most people with the disorder (high sensitivity) and correctly excludes most people without the disorder (high specificity).

We were able to find only one review "Clinicians' Compendium Of Assessment Tools for Mental Health Clients from Culturally and Linguistically Diverse Backgrounds [7]" done in Australia that has reviewed validation status of questionnaires available in languages other than English. While the Compendium does list a few assessment tools that have been translated into Urdu, Urdu was not a search term in that review and all instruments in Urdu were included in the category of Instruments in Languages Other Than English (LOTE) for which insufficient published information was available and accessible. In this review we have therefore, tried to explore how many questionnaires measuring psychiatric symptoms are available in Urdu, whether indigenous or translated, that have undergone some degree of validation. We have also tried to assess to what extent these questionnaires have undergone either criterion validation (applicable to all questionnaires) or cross-cultural validation (applicable to translated questionnaires only).

## Methods

### Search strategy

We searched Medline (since 1951), Embase (since 1974) and PsycINFO (since 1806) through the  website. We searched  (a website that indexes most of the medical journals published in Pakistan including those that are not indexed on Medline or Embase) on 8 February 2006 using the following search terms; Urdu psychiatric rating scale, and Urdu and Psychiatry. We searched the references, and the references of the references, of the retrieved articles. We contacted 21 psychiatrists and four psychologists working in Pakistan, and one psychiatrist working in UK, to find out if they were aware of any scales validated in Urdu that were not on our list. We searched the titles of all the dissertations, and in 2 cases full dissertations, in the subject of Psychiatry submitted to the College of Physicians and Surgeons Pakistan.

### Inclusion/exclusion criteria

Only those studies that reported the process of assessment of either criterion validity or cross-cultural validity of a questionnaire measuring psychiatric symptoms in Urdu language were included.

Studies were excluded if they reported use of a questionnaire in Urdu but did not provide any details about validation. Similarly, studies reporting validation of questionnaires in Urdu for uses other than measuring psychiatric symptoms were not included.

### Analysis

One of us (SA) extracted validation data from all the studies except two [8,9]. The data from these two studies was extracted by RAF. We used the following parameters.

For ***criterion validation ***we extracted data on all the questionnaires about the setting they had been validated in, sample size, the gold standard used, reliability values, area under the curve, and validity coefficients like sensitivity, specificity, positive predictive value, negative predictive value, and overall misattribution ratio.

Guillemin et al (1993) [10] have suggested the following guidelines to preserve equivalence in adapting measures developed in one language and culture for use in another language and culture; 1. more than one independent translations, 2. as many back-translations as translations[11], 3. a committee approach to produce a final version in the target language, and 4. pre-testing to establish equivalence in source and target versions using either a probe technique (using qualitative methods) or bilingual method (administering both the versions to a group of bilingual lay people to assess if they respond similarly to the same question in both languages).

For ***cross-cultural validation ***we, therefore, extracted data on process of back-translation, whether or not a committee approach had been taken, and whether the authors had done pre-testing. If a bilingual approach for pre-testing had been taken we assessed whether the authors had examined linguistic equivalence (whether the questionnaire has been translated literally), conceptual equivalence (whether the translation captures the meaning of the original), and scale equivalence (whether both the source and target language versions identify the same individuals as high scorers) [12].

For being clinically useful, besides being valid, a new test or scale must also be ***reliable***. The reliability of a test describes the degree to which the test consistently measures a variable [13]. The higher the reliability of a test the more likely it is that the test will yield a similar result when administered; by different raters (inter-rater reliability), by the same rater after some interval of time (intra-rater reliability), or in two halves (split-half reliability), and that items measuring different dimensions of the same phenomenon will be scored similarly (internal consistency). A scale can be reliable but not valid, but if a scale is unreliable it can not be valid. We therefore extracted data on different forms of reliability whenever it was reported in a paper.

## Results

Our initial databases search yielded 29 studies. Of these 15 were found to be relevant. Our secondary search yielded 42 more studies. Three of these were found through the experts we had contacted. Of these 17 were found to be relevant. Thus a total of 32 studies, reporting either cross-cultural or clinical validation of 19 psychiatric questionnaires in Urdu were included in the review. Details of validation of translation of Edinburgh Postnatal Depression Scale were found in the abstract of validation study of Harvard Trauma Questionnaire [14].

### Indigenously developed scales

We found six rating scales which did not have an original English language version. Three of these the Aga Khan University Anxiety and Depression Scale (**AKUADS**) [15-18], Pakistan Anxiety and Depression Questionnaire (**PADQ**) [19], and Siddiqui-Shah Depression Scale (**SSDS**) [20,21] were developed indigenously in Urdu, while Bradford Somatic Inventory (**BSI**) [22-27] was developed simultaneously in Urdu and English.

Acute Stress Reaction Questionnaire (**ASR-Q**) [8,28] and Post Traumatic Stress Disorder Questionnaire (**PTSD-Q**)[9] were developed converting DSM-IV diagnostic criteria for these disorders into questions in English language which were then translated into Urdu. We have included these with indigenously developed questionnaires as there is no equivalent questionnaire in English. However, as these were developed translating DSM criteria these are not truly indigenous scales.

### Translated scales

Thirteen scales were translated from English and underwent some degree of validation. These included Clinical Interview Schedule-revised (**CIS-R**) [29], Eating Attitudes Test (**EAT**) [30], Edinburgh Postnatal Depression Scale (**EPDS**) [14], General Health Questionnaire (**GHQ**) both 12 [31] and 28 [12] items versions, Harvard Trauma Questionnaire (**HTQ**) [14], Hospital Anxiety and Depression Scale (**HADS**) [32,33], **How I Feel **scale [18], Hopkins Symptoms Checklist (**HSCL**)[34], Inventory of Traumatic Grief (**ITG**)[35], Personal Health Questionnaire (**PHQ**) [36-38], Strengths and Difficulties Questionnaire (**SDQ**) [39], Self-Reporting Questionnaire (**SRQ**) [22,25,36,38,40,41], and World Health Organization Quality of Life scale Brief version (**WHO-QOL BREF**)[42].

### Cross-cultural Validation status

Table 1 shows the cross-cultural validation status for all the scales translated from English language. Of the thirteen translated scales six, the CIS-R, EAT, HADS, How I feel scale, SRQ AND WHO-QOL BREF were evaluated most rigorously for cross-cultural validation employing back-translation, translation committee, and pre-testing in a non-clinical sample. The GHQ-28 did not have back-translation done but had a translation committee and was also pre-tested.

**Table 1:** Cross-cultural validation status of all the psychiatric rating scales in Urdu that have been translated from English language. (BSI was developed simultaneously in Urdu and English)

**Scale**	**Study**	**Back-translation done**	**Translation committee**	**Pre-testing done**	**Validation Population**	**Linguistic Equivalence evaluated**	**Conceptual Equivalence evaluated**	**Scale Equivalence evaluated**
**CIS-R**	[29]	YES	YES	Probe technique	Three women	No	No	No
**EAT**	[30]	YES	YES	YES, bilingual method	College students	Yes	Yes	Yes
**EPDS**	[14]	NR	NR	NO	N/A	No	No	No
**GHQ 12**	[31]	NO	YES	NO	N/A	No	No	No
**GHQ-28**	[12]	NO	YES	Yes, Bilingual method	Medical & nursing students	Yes	Yes	Yes
**HADS**	[32, 33]	YES	YES	YES, bilingual method	Medical students	Yes	Yes	Yes
**How I Feel**	[18]	YES	YES	YES, probe technique	Pregnant women	No	No	No
**HSCL**	[34]	YES	YES	NO	N/A	No	No	No
**HTQ**	[14]	YES	YES	NO	N/A	No	No	No
**ITG**	[35]	YES	NR	NO	N/A	No	No	No
**PHQ**	[36, 38]	NR	NR	NR	N/A	No	No	No
**SDQ**	[39]	YES	YES	NO	N/A	No	No	No
**SRQ**	[25, 36, 38, 40, 41]	YES	YES	YES, probe technique	8 mothers from the study area	No	No	No
**WHO-QOL BREF**	[42]	YES	YES	Bilingual method	Healthy bilingual subjects	Yes	Yes	Yes

N/A = Not applicable, NR = Not reported

The EAT, GHQ-28, HADS and WHO-QOL-BREF were pre-tested tested using the bilingual method and had their linguistic, conceptual and scale equivalence examined. The CIS-R, How I Feel scale and SRQ were pre-tested using the probe technique. The SDQ was back-translated and had a translation committee but was not pre-tested on a non-clinical sample. The EPDS, GHQ-12 and PHQ did not undergo cross-cultural validation.

### Reliability and Criterion validity coefficients

Additional file 1 shows the reliability and validity coefficients for the 12 questionnaires, indigenous or translated, that have been evaluated for criterion validity in a clinical sample. Among the indigenous questionnaires the AKUADS, ASR-Q, BSI (44, 21 and 14 items versions), PADQ, and PTSD-Q were examined for criterion validity. Among the translated questionnaires GHQ (12 items version), HADS, How I Feel scale, PHQ, SDQ, SRQ and SSDS underwent criterion validity evaluation.

The AKUADS, BSI and SRQ were the ones that were most extensively evaluated for criterion validity.

### Gold standards used

The gold standards against which the new scales were validated were Psychiatric Assessment Schedule (PAS) [43] in five studies, Psychiatrists' Clinical Diagnoses and ICD-10 Research Diagnostic Criteria in four studies each, DSM-IV criteria applied by psychiatrists and Diagnostic Interview Schedule used in two studies each, and DSM-III-R criteria and Clinical Interview Schedule used in one study each. There are several mentions of the instruments used being translated in Urdu but none of these gold standards has itself been validated in Urdu.

### Quality of reviewed studies

Quality of included studies varied greatly. Some studies had very small sample sizes like 20 for HADS or 30 for PTSD-Q validation study making it questionable if the results could be extrapolated to the whole Pakistan population or even a sub-population. Four studies have used "Psychiatrists' Clinical Diagnoses" as gold standard [15,17,20,39] rather than using a more valid gold standard like a structured or semi-structured diagnostic interview. This puts the validity of the validation itself in question. Many studies have either not mentioned Reliability at all or mentioned that they tested for Reliability but have not provided any values, as detailed in Additional file 1.

## Discussion

To our knowledge our study is the first of its kind looking at the validation status of all the psychiatric rating scales available in Urdu. We found 19 rating scales, 6 indigenous and 13 translated from English, which have undergone some degree of validation in Urdu. Among the six indigenous scales, the BSI has been most extensively validated both in urban and rural settings. Among the other indigenous scales AKUADS, PADQ and SSDS were validated in reasonably large samples. ASR-Q did not go through a criterion validation study while the PTSD-Q validation study had a very small sample size.

Among the 13 translated scales only the How I Feel scale, the SDQ and SRQ were evaluated for *both *cross-cultural and criterion validation, the SRQ being the most extensively evaluated and validated. Rest of the translated scales underwent either only cross-cultural (CIS-R, EAT, GHQ-28, HSCL, HTQ, ITG, WHO-QOL) or criterion (GHQ-12, PHQ) validation. The HADS scale underwent both cross-cultural and criterion validity evaluation but these were two different translations one undergoing the former and the other the latter.

The Australian "Clinicians' Compendium Of Assessment Tools for Mental Health Clients from Culturally and Linguistically Diverse Backgrounds [7]" shows that BDI-II, HADS, EPDS and GHQ have all been translated and undergone some degree of validation in Arabic; EPDS, GHQ and HADS in Italian; BDI, EPDS, GHQ and HADS in Chinese/Cantonese; and BDI-II and HADS in Spanish. As explained above Urdu was not a search term in this review.

On one hand it was rather surprising and encouraging to find 19 questionnaires measuring psychiatric symptoms in Urdu which had undergone some degree of either cross-cultural or criterion validation. On the other hand most of these are screening tools for anxiety, depression or general psychiatric morbidity. The very commonly used research tools like HRSD, MADRS, BDI, PANSS etc, and the definitive diagnostic instruments like SCID have *not *undergone any sort of validation in Urdu.

Bhui et al. [44] have suggested that even within a broad ethnic group expressions of distress may vary between different sub-groups and may change as a result of acculturation. The GHQ-12 performed better than the ADI (Amritsar Depression Inventory, developed in the Punjab in India) in detecting depression even in the Punjabi population settled in UK. This suggests that even instruments developed in one language may not be equally valid for all sub-groups speaking that language depending on the culture they are living in. In that sense language and culture are not one and the same where validation of instruments is concerned.

So what does one do when one wants to do research in a language other than English and there is no fit-for-purpose tool that has been validated in that language? In their review of cross-cultural adaptation of health-related quality of life measures Guillemin et al. [10] have stated that there are two possible options. The first is to develop a new measure using culturally defined, within-group variables that have been developed and described in terms of the language and customs of a particular culture at a particular time, called the Emic approach [4]. The second approach is to use a measure from another language and culture applying the concepts of behaviour and techniques of measuring that behaviour from the so-called source culture to the target culture, called the Etic approach.

The problem with an exclusively emic approach is that it does not allow quantitative comparison across times and between cultures. The problem with an exclusively etic approach is that manifestations and expressions of a universal phenomenon, for example depression, may be different in different cultures, and thus may be missed if concepts and measures from one culture are applied blindly to another culture [4]. The first is time, labour and expertise intensive because of the need to conceptualise a new measure and select its items, while the second is fraught with the difficulties of the relevance and validity of a measure developed in one language and culture being used in another language and culture.

In Urdu it seems like both approaches have been used, with most scales being translated from English and a few being developed indigenously from complaints of Pakistani patients later diagnosed as suffering from Depression and Anxiety. However, since even the latter were validated against etic constructs like ICD and DSM diagnoses it is difficult to say if there are any purely emic instruments in Urdu. This raises the question whether there should be a different set of criteria for diagnosing depression in Pakistan if people suffering from depression in Pakistan present with different expressions of distress compared to patients in the West? If the diagnostic criteria are different should we call this syndrome something other than depression? Questions like these would only be answered after a lot more cultre-centred research than has been carried out as yet.

## Conclusion

Nineteen questionnaires measuring psychiatric symptoms have so far been evaluated for cross-cultural and/or criterion validity in Urdu. Six of these have been developed indigenously while thirteen have been translated from English. The BSI, SRQ and AKUADS are the questionnaires that have been most thoroughly evaluated in Urdu.

## Competing interests

The author(s) declare that they have no competing interests.

## Authors' contributions

SA and RAF thought of the idea. SA developed the protocol. SA and RAF carried out the searches. SA, RAF and AA contributed to retrieving the data. All authors reviewed the manuscript critically and approved it.

## Pre-publication history

The pre-publication history for this paper can be accessed here:



## Supplementary Material

Additional file 1 Reliability and validity coefficients of psychiatric rating scales in Urdu that have undergone evaluation of criterion validity. The file contains data for reliability, and validity coefficients like sensitivity, specificity, positive predictive value and negative predictive value for scales that have been validated against a gold standard in a clinical sample Click here for file
