# Supplementary material for: Psychiatric rating scales in Urdu: a systematic review
Source: BMC Psychiatry. 2007 Oct 26;7:59. doi: 10.1186/1471-244X-7-59 (PMC2186305; doi:10.1186/1471-244X-7-59)
Supplement: Additional file 1 — Reliability and validity coefficients of psychiatric rating scales in Urdu that have undergone evaluation of criterion validity. The file contains data for reliability, and validity coefficients like sensitivity, specificity, positive predictive value and negative predictive value for scales that have been validated against a gold standard in a clinical sample [file 1471-244X-7-59-S1.doc]

**Additional file 1. Reliability and validity coefficients of psychiatric rating scales in Urdu that have undergone evaluation of criterion validity**

| **Scale** | **Study** | **Setting** | **Sample size** | **Gold standard** | **Reliability evaluated** | **Cut-off score** | **Area under ROC Curve** | **Sensitivity(%)** | **Specificity(%)** | **PPV (%)** | **NPV (%)** | **OMR (%)** |
| --- | --- | --- | --- | --- | --- | --- | --- | --- | --- | --- | --- | --- |
| **AKUADS** | [15] | Psychiatric outpatients in Karachi, Pakistan | 61 | Psychiatrists’ clinical diagnoses | 22 items showed item-item correlation ≥ 0.75 | 19/20 | NR | 66 | 79 | 83 | 60 | NR |
|  | [16]  [17] | Urban squatter settlement in Karachi, Pakistan | 487 | Psychiatrists’ clinical diagnosis | Item-item correlation 0.2-0.62, item-total correl. 0.29-0.71 | 19/20 | NR | 74 | 81 | 63 | 87 | 21 |
|  | [18] | Antenatal clinic attendees in Hyderabad, Pakistan | 200 | DSM-IV criteria applied by psychiatrist | Cronbach’s alpha=0.87, mean item-total correlation=0.42 | 31.5 | 0.73 | 63 | 72 | NR | NR | NR |
| **ASR-Q** | [8, 28] | Villager near Khanewal, Pakistan | 62 | Not clearly mentioned | Inter-rater reliability mentioned but no details given | NR | NR | NR | NR | NR | NR | NR |
| **BSI-44** | [24] | Urban community in Rawalpindi, Pakistan | BSI-44=760, ICD-10 RDC-220 | ICD-10 research Diagnostic Criteria | Overall item agreement between raters=95% | 20/21 | NR | 65 | 97 | 94 | 89 | NR |
|  | [22] | Susral village in Punjab, Pakistan | 664 = BSI, 191 = PAS | ICD-10 Diagnostic Criteria for Research | Overall BSI item-agreement between raters over 90% | 25/26 | NR | 87 | 61 | NR | NR | NR |
|  | [25] | Susral village in Punjab, Pakistan | BSI= 628, PAS= 191 | PAS | Overall 90% agreement for items between raters | Women 20/22  Men 11/14 | NR | 82  59 | 71  62 | 95  44 | NR | NR |
| **BSI-44**  **BSI-21**  **BSI-14** | [26] | Medical clinic attenders in a tertiary care hospital in Lahore, Pakistan | 55 | DSM III-R criteria | NR | 27/28  13/14  9/10 | 0.821  0.830  0.817 | 77  87  67 | 71  75  75 | 60  56-60  NR | NR  NR  NR | NR  NR  NR |
| **BSI-21** | [27] | Mountain villages in Chitral, Pakistan | 515 | ICD-10 Diagnostic criteria for research | Overall item agreement between raters = 96.5% | 20/21  13/14 | NR | 80  71 | 77  100 | NR  NR | NR  NR | NR  NR |
| **GHQ-12** | [31] | Primary care attenders in Gujar Khan, Pakistan | GHQ-12 = 294, PAS = 238 | PAS | NR | 1/2 | 0.91 | 93 | 88 | 65 | NR | NR |
| **HADS**  **Anxiety S.S.**  **Depression S.S.** | [33] | Urdu-speaking psychiatric outpatients, Bradford, UK | 20 | CIS | NR | 7/8 | NR | 66  85 | 37  70 | NR | NR | NR |
| **HOW I FEEL** | [18] | Antenatal clinic attendees in Hyderabad, Pakistan | 200 | DSM-IV criteria applied by psychiatrist | Cronbach’s alpha 0.92, mean item-total correlation 0.52 | 83.5 | 0.74 | 74 | 69 | NR | NR | NR |
| **PADQ** | [19] | Inpatients & outpatients in Lahore, rural community clinics, Pakistan | 330 | ICD-10 diagnostic criteria for research | NR for full scale,  Alpha for AD scale = 0.92  Alpha for D scale = 0.91 | 11/12 | NR | 95 | 91 | 91 | 95 | 8 |
| **PHQ** | [36] | Mandra village in Pakistan | 258 | PAS | NR | 5/6 | NR | 69.6 | 85.2 | 78.9 | 77.8 | 21 |
|  | [38] | Mandra village in Pakistan | PHQ= 259, PAS= 103 | PAS | NR | ≥6 | NR | 82 | 74 | 79 | 78 | NR |
| **PTSD-Q** | [9] | Rural Community outside Karachi, Pakistan | 30 | Diagnostic Interview Schedule | Mentions Inter-rater reliability but gives no details | NR | NR | 100 | 90 | 81 | 100 | 6.66 |
| **SDQ**  **(Total difficut. score)** | [39] | Child psychiatry clinics in Karachi, Pakistan | 72 cases, 140 controls | Clinical assessment by child psychiatrist | NR | 17.5 | 0.77 | 69 | 71 | NR | NR | NR |
| **SRQ** | [36] | Mandra village in Pakistan | PHQ= 259, PAS= 103 | PAS | NR | 5/6  8/9 | NR | 93.1  67.0 | 80.8  91.8 | 77.9  86.3 | 94.1  78.4 | 16.6  17.6 |
|  | [40] | Mandra village in Pakistan | SRQ= 773, PAS ~ 263 | PAS | NR | 4/5 | NR | 63 | 77 | 47 | 85 | NR |
|  | [25] | Susral village in Punjab, Pakistan | SRQ =628, PAS= 191 | PAS | Overall 90% agreement for items between raters | Women 7/8  Men 3/4 | NR | 78  78 | 81  70 | 95  62 | NR  NR | NR  NR |
|  | [38] | Mandra village in Pakistan | SRQ= 259, PAS= 103 | PAS | NR | ≥9 | NR | 80 | 85 | 86 | 79 | NR |
|  | [22] | Susral village in Punjab, Pakistan | SRQ =664, PAS= 191 | ICD-10 Diagnostic Criteria for Research | Overall item agreement between raters over 90% | 5/6 | NR | 85 | 70 | NR | NR | NR |
| **SSDS** | [21] | University students, psychiatric outpatients and inpatients | 266 | Psychiatrists’ clinical diagnosis | Yes | 26/27  37/38  50/51 | NR | 95  77  50 | 55  84  94 | 38  58  70 | 97  93  87 | 36  18  16 |
|  | [20]  [35] | Psychiatric patients and controls | Pts = 47, controls = 36 | DSM-IV | NR | 36/37 | NR | 79 | 89 | NR | NR | NR |

**NR** = Not reported, **NPV**= Negative Predictive Value, **OMR** = Overall Misattribution rate, **PAS** = Psychiatric Assessment Schedule, **PPV** = Positive Predictive Value,
